# A Study on Influence of Minivan Front-End Design and Impact Velocity on Pedestrian Thorax Kinematics and Injury Risk

**DOI:** 10.1155/2018/7350159

**Published:** 2018-09-03

**Authors:** Fang Wang, Chao Yu, Guibing Li, Yong Han, Bingyu Wang, Jikuang Yang, Diandian Lan

**Affiliations:** ^1^School of Mechanical and Automotive Engineering, Xiamen University of Technology, Xiamen, China; ^2^Fujian Collaborative Innovation Center for R&D of Coach and Special Vehicle, Xiamen, China; ^3^School of Mechanical Engineering, Hunan University of Science and Technology, Xiangtan, China; ^4^Research Center of Vehicle and Traffic Safety (VTS), State Key Laboratory of Advanced Design and Manufacturing for Vehicle Body, Hunan University, Changsha, China; ^5^Department of Applied Mechanics, Chalmers University of Technology, Gothenburg, Sweden

## Abstract

Thoracic injuries occur frequently in minivan-to-pedestrian impact accidents and can cause substantial fatalities. The present research work investigates the human thoracic responses and injury risks in minivan-to-pedestrian impacts, when changing the minivan front-end design and the impact velocity, by using computational biomechanics model. We employed three typical types of minivan model of different front-end designs that are quite popular in Chinese market and considered four impact velocities (20, 30, 40, and 50 km/h). The contact time of car to thorax region (CTCTR), thorax impact velocity, chest deformation, and thoracic injury risks were extracted for the investigation. The results indicate that the predicted pedestrian kinematics, injury responses, and thoracic injury risks are strongly affected by the variation of the minivan front-end design and impact velocity. The pedestrian thoracic injury risks increase with the increasing vehicle impact velocity. It is also revealed that the application of the extra front bumper is beneficial for reducing the thoracic injury risk, and a relatively flatter minivan front-end design gives rise to a higher thoracic injury risk. This study is expected to be served as theoretical references for pedestrian protection design of minivans.

## 1. Introduction

Pedestrians are typical vulnerable road users (VRU) in road traffic accidents, who sustain extremely high injury risk [1–3]. As reported in the literature, over 270,000 pedestrians died in road traffic accidents every year, and the pedestrians accounted for as much as 22% of the traffic accident fatalities of the world [4] and even 45–55% in some developing countries [5]. In China, a number of studies showed that pedestrian impact accidents were the second largest proportion in terms of both the accident types and deaths and injuries involved in all types of traffic accidents [6–9]. In developing countries with wide rural areas like China, the occupancy of minivans is far more than that in developed countries in view of the advantages of large passenger carrying capacity, low price, and low fuel consumption. Minivans started in the 1980s in China and are extremely popular and widely used in countryside. Particularly, the implementation of the “car to the countryside” policy in 2009 promoted the sharp increase of minivans. It was reported that over 30,000 minivans involved accidents in 2012 resulted in 6865 deaths and around 35,000 injured in China, making up 11.0%, 9.4%, and 11.1% of the total amounts, respectively [10, 11]. Furthermore, given the relatively weak awareness of traffic safety of the road users in rural areas, the growing number of minivans in China will inevitably lead to the increase of minivan-related accidents.

Human head and lower extremity were the body regions most frequently injured in pedestrian impact accidents, which have caused extensive concerns as identified in related studies [12–16]. However, real-world accident data have shown that thoracic injuries are especially important in minivan-to-pedestrian collisions since the minivan bonnets are significantly higher than those of small passenger cars and the initial impact on the thorax is more severe [17]. Moreover, thoracic injuries were recognized to be the second most common injuries to cause fatalities right after head injuries in the research of Hu and Klinich [18] and Martin et al. [19]. Through the analysis on 839 fatal injuries involved pedestrian impact accidents, Fildes et al. [20] found that 50% of pedestrian deaths were resulted from the AIS4+ injuries of the head/chest or both, and 17% of the fatalities were solely caused by thoracic injury. Chidester and Isenberg [21] analyzed 527 pedestrian impact accidents, 172 out of which were light trucks to pedestrian impacts, and they concluded that in all AIS2+ injuries, thoracic injuries accounted for a proportion only lower than the head and lower extremity injuries. Li et al. [22] analyzed pedestrian injuries in collisions with minivans and sedans using the accident data and found that thoracic injuries accounted for 24% of all AIS3+ injuries in minivan cases, which was the double of that in sedan cases.

However, thoracic injuries have not attracted enough research concerns and no test procedure exists [19, 23]. From the perspective of injury biomechanics, quite few studies have been conducted, and through those research it was confirmed that the injury patterns and injury mechanisms from box-shaped vehicles differ from bonnet-front vehicle collisions [16, 24], and flat-front vehicles would produce a higher probability of thoracic injury than the bonnet-front vehicles [25]. Similar findings were also reported in the study of Mizuno and Kajzer [26] where they found that pedestrian chest acceleration in single-compartment vehicle impacts was higher than that in impacts of other vehicles, and the study of Han et al. [27] who found that one-box vehicle tended to lead to higher pedestrian thoracic injury risk than other types of vehicles (medium-size sedan, minicar, and sport utility vehicle SUV). Nevertheless, the previous studies were prone to focus on the comparison of minivan with other vehicle types, while no attempt has been made to specifically analyze how minivan front design would affect the predicted thoracic injury biomechanical responses of the pedestrian during an event of minivan-to-pedestrian impact.

Therefore, the purpose of the current study is to investigate the thoracic kinematics, injury responses, and injury risks when struck by minivan fronts of different designs at different impact velocities via numerical modelling of minivan-to-pedestrian frontal impacts using a previously developed and validated pedestrian computational biomechanics model. The findings of this study are expected to serve as references for minivan front-end structure design in terms of pedestrian thoracic injury prevention.

## 2. Materials and Methods

### 2.1. Model Description

#### 2.1.1. Pedestrian Model

**Figure 1:**
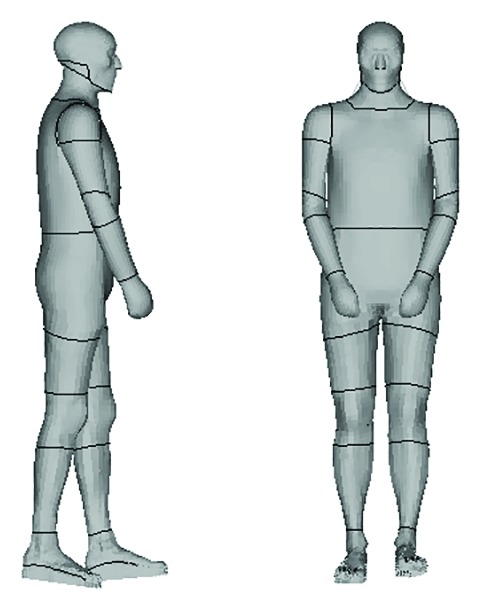
The HNU-HBM pedestrian finite element model used in this study.

The pedestrian model of an existing HNU-HBM (Hunan University Human Body Model) was employed in our study (see Figure 1) using the LS-DYNA nonlinear finite element code [28]. This model has been validated individually in terms of body components (head, neck, torso, and lower extremity) [29–35] as well as for the whole body motion against a reported cadaver test [36]. Particularly, the HNU-HBM thorax model was verified and applied by the authors in a previous research [32]. In total, the pedestrian model consists of over 273,000 elements and 215,000 nodes.

#### 2.1.2. Minivan Models

**Figure 2:**
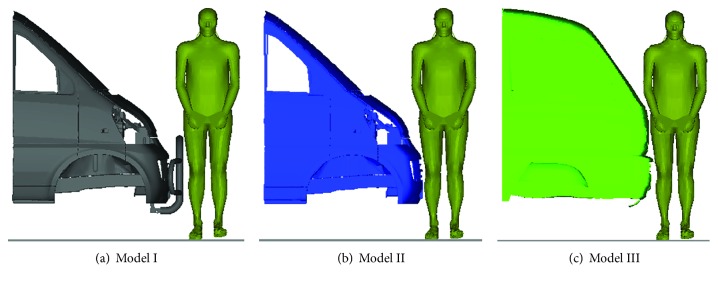
Schematic of the three minivan models used for the pedestrian impact simulation in this study.

To investigate the thoracic responses in frontal impact with different types of minivan, three minivan finite element (FE) models of typical front-end designs in Chinese market were employed, which have been previously developed and validated in detail against crash experimental data [37, 38]. In the validations, the vehicle deformation and acceleration response curves of the models showed good agreements with the experimental data in 100% rigid barrier (RB) crash tests at 50 km/h. For time efficiency in the simulation, only the front-end components and adjacent part of the passenger compartment were extracted from the full FE vehicle models in the current study, see Figure 2. Model I and model II have the same front-end designs, and the only difference between them is with/without the extra bumper at the front (see Figures 2(a) and 2(b)). The major difference of model III from the other two models is in the overall shape, such as the height of bonnet leading edge and slope of windshield (see Figure 2(c)). The selection of these models was defined to cover the most popular versions and the range of minivan front-end designs in Chinese market.

### 2.2. Boundary Conditions of the Minivan-to-Pedestrian Impacts

**Figure 3:**
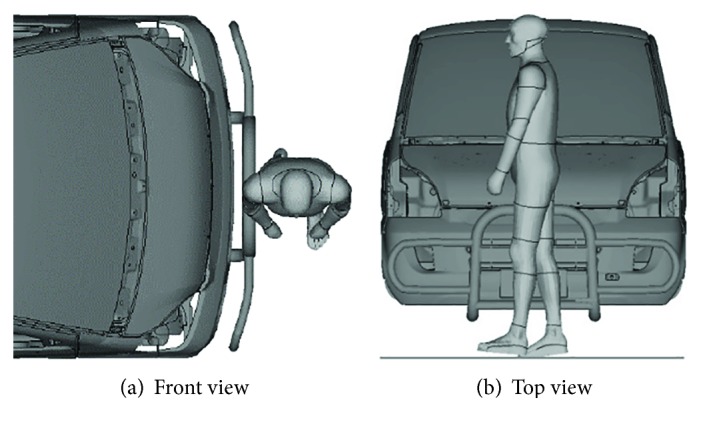
Description of the minivan-to-pedestrian impact simulation scenario.

It has been identified that about two-thirds of pedestrians were hit from the side by a vehicle when walking across a road in real-world accidents [21, 39]; thus, the pedestrian model was set in a walking posture with the right side being toward the center of a given minivan model in the simulations, see Figure 3. Four impact velocities (20, 30, 40, and 50 km/h) were chosen for the simulations, as about 90% of car-to-pedestrian impact accidents occurred below 50 km/h, and the fatality risk of the pedestrian rose sharply when the impact velocity increases from 20 km/h to 50 km/h [14, 40].

Thus, in total, 12 simulations were conducted, and the thoracic kinematics and injury parameters of the pedestrian model were extracted from the simulations for a comparative study of pedestrian thoracic injury biomechanical responses.

### 2.3. Thorax Kinematics and Injury Biomechanical Parameters

#### 2.3.1. Thorax Kinematics Parameters

*The contact time of car to thorax region (CTCTR)* is defined as the time from the initial contact (usually the contact between the lower extremity and the vehicle for a typical car-to-pedestrian impact accident) to the moment when thorax firstly contacts with the vehicle exterior. CTCTR was used in the current study as a parameter to assess the pedestrian kinematics, since it helps to determine the level of thorax impact velocity.

*Thorax impact velocity,* that is, the resultant translational velocity of the pedestrian thorax relative to the vehicle exterior at the moment of CTCTR, has been identified to be a direct factor in the determination of the thoracic injury responses [41]. Therefore, it has been chosen for pedestrian kinematics analysis.

#### 2.3.2. Thoracic Injury Parameter and Injury Risk

*Chest deformation,* defined as the maximum difference between the initial and minimum left-right distance of the deformed thorax contours in transverse plane in side impact, is a critical injury parameter to evaluate thoracic injury severity/risk in vehicle safety assessment as well as the research field of human thoracic injury biomechanics under impact loading conditions [42, 43]. In the current study, the chest deformations have been analyzed for all the simulation cases.

*Thoracic injury risk:* various injury criteria have been proposed in the literature for the evaluation of human thoracic injury risk/severity in impact loading conditions, some of which were used in legal regulations of vehicle crash safety performance in terms of occupant injury prevention. TTI (thoracic trauma index) was a widely used thoracic injury criterion (a standard thoracic injury index employed in the US regulation for assessing side impact crashworthiness and occupant injury prevention), which was developed based on a series of side PMHS (postmortem human subject) experiment data carried by Eppinger et al. [44] and accounted for both upper and lower rib accelerations of the human thorax as well as the age and mass factors of the human body. Since TTI was identified to be a good predictor of thoracic injury severity in our previous study [33], it was applied in the current research to evaluate the thoracic injury risk of the pedestrian when struck by three minivan models at different velocities. Then, the predicted TTI values were related to specific thoracic injury risks of an AIS (abbreviated injury scale, a commonly used injury code designed to quantify the severity of the injury to human body, lowest AIS0 means no injury, and highest AIS6 means currently untreatable injury) level based on the previously established injury risk functions/curves which illustrated the relationships between TTI and injury severity [42].

## 3. Results

### 3.1. Pedestrian Kinematics

**Figure 4:**
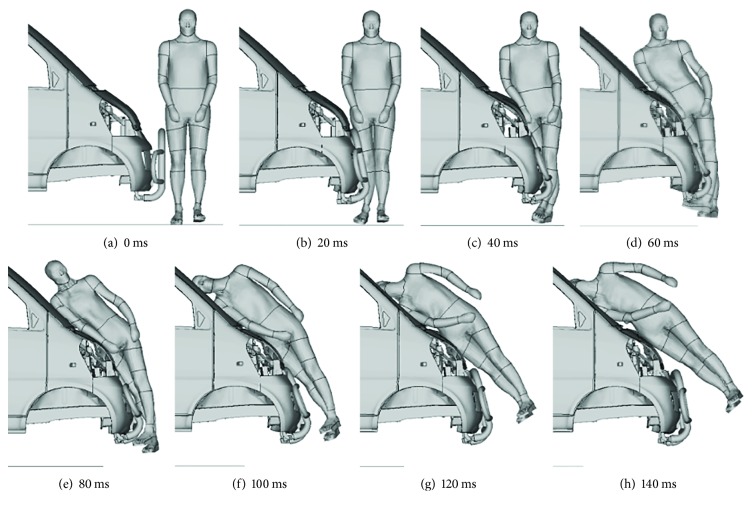
Pedestrian kinematics responses when struck by model I at 50 km/h.

Figure 4 demonstrates the typical dynamic responses of the pedestrian at different moments from 0 ms to 140 ms during an impact with the model I at 50 km/h (with other cases not shown). During the impact, the pedestrian rotated along the frontal surface of the minivan, and the contact between the vehicle and different human body regions including lower extremity, pelvis, abdomen, thorax, and head was observed, successively.

#### 3.1.1. Contact Time of Car to Thorax Region (CTCTR)

**Figure 5:**
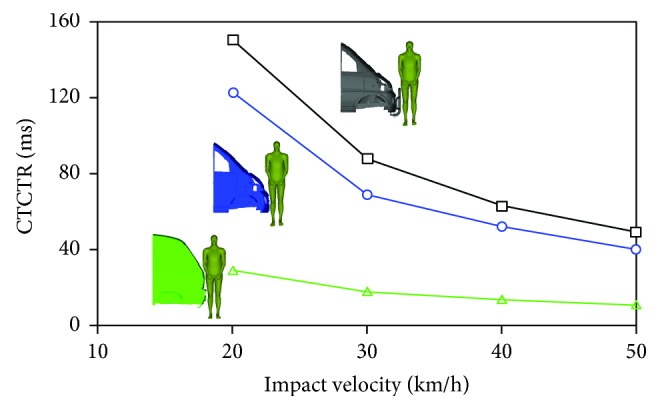
Comparison of the contact time of car to thorax region (CTCTR) of the pedestrian when struck by the three minivan models at different impact velocities.

The relationships between the CTCTR and the impact velocity for the three minivan models were built, as shown in Figure 5. It could be observed from Figure 5 that both the front-end design of the minivan and impact velocity had significant effects on CTCTR. For model I and II, the pedestrian thorax contacted the vehicle exterior later than model III, as the distances between the pedestrian thorax and the vehicle were relatively longer than those in model III cases. Such trend for model I was more obvious than model II due to the additional front bumper, which led to the highest CTCTR in model I cases. Model III, with the relatively flatter frontal shape, presented the lowest level of CTCTR.

With regard to the effects of the vehicle velocity, differences in the CTCTR distribution for the three minivan models were found when changing the level of the vehicle velocity from 20 km/h to 50 km/h, see Figure 5. Specifically, increasing the vehicle velocity would weaken the effects of the variation of vehicle front-end design, and no predominant effects were found when increasing the vehicle impact velocity for model III, comparing with the remaining minivan models.

#### 3.1.2. Thorax Impact Velocity

**Figure 6:**
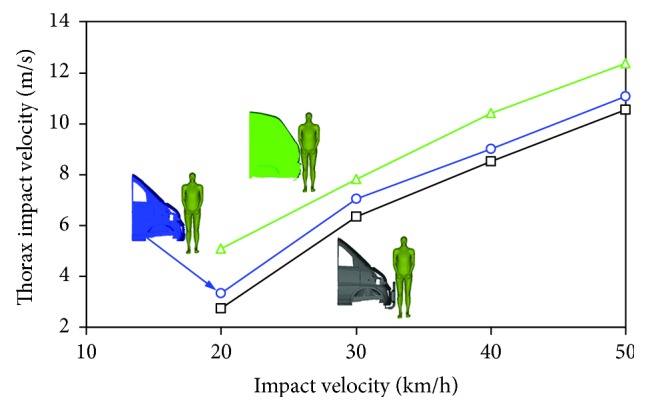
Comparison of the thorax impact velocity of the pedestrian when struck by the three minivan models at different impact velocities.

Figure 6 shows the distribution of pedestrian thorax impact velocity when struck by the three minivan models at different vehicle velocities. The vehicle velocity appeared to exert more predominant effects on thorax impact velocity than the front-end design of the minivan did. No big difference in thorax impact velocity was found among different vehicle front-end designs, which is not in line with the CTCTR case where evidently different curve tendency was found for different minivan models (see Figure 5).

### 3.2. Chest Deformation

**Figure 7:**
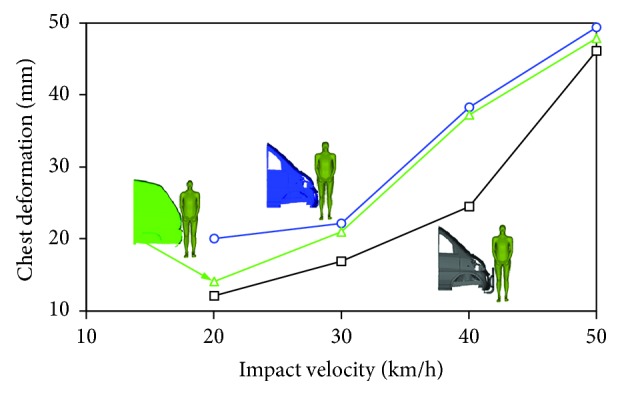
Comparison of the chest deformation of the pedestrian when impacting with the three minivan models at different impact velocities.

The chest deformations of all the impact simulations when varying both minivan model and vehicle impact velocity were described in Figure 7. The chest deformation noticeably increased with increasing vehicle impact velocity. In contract with the analysis focusing on CTCTR and thorax impact velocity, the chest deformation values of the pedestrian in impacts with model II and model III were pretty close to each other for almost all the vehicle velocity levels with the exception of 20 km/h and were clearly higher than those with model I. In impacts lower than 30 km/h, the calculated chest deformation of the pedestrian was quite mild (lower than 22 mm), but for higher speed impacts (>30 km/h), a significant increase of the chest deformation was observed, especially for model II and model III. It should be noted that the peak chest deformation value for model II reached up to 48 mm that exceeded the critical value (42 mm) required by the standard regulation in Europe [45].

### 3.3. Thoracic Injury Risk

**Figure 8:**
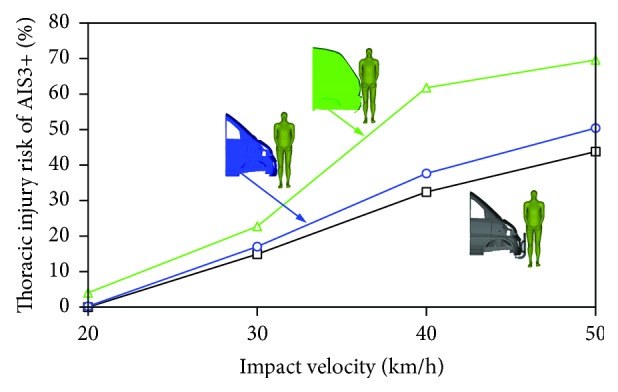
Comparison of the thoracic AIS3+ injury risks of the pedestrian when struck by the three minivan models at different impact velocities.

**Figure 9:**
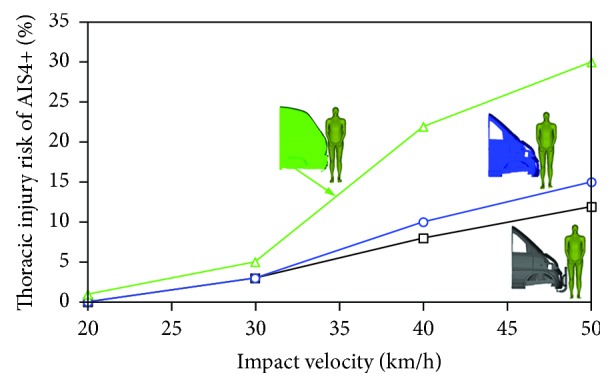
Comparison of the thoracic AIS4+ injury risks of the pedestrian when struck by the three minivan models at different impact velocities.

The calculated TTI values of all the simulation cases were collected to demonstrate the effects of minivan front-end design and impact velocity on pedestrian thoracic injury risks, see Table 1. The specific thoracic injury risks for AIS3+ and AIS4+ obtained through the method described in Section 2.3 were measured and compared with each other in terms of minivan front-end design and vehicle impact velocity (Figures 8 and 9). From these two figures, measurable effects of both the vehicle front-end design and the impact velocity were found on the predicted thoracic injury risks. Particularly, for minivan model I and model II, the effects of the variation of vehicle impact velocity appeared to be more important than changing from one minivan model to another. For minivan model III, more significant increase in both AIS3+ and AIS4+ thoracic injury risks were observed to be resulted from increasing the vehicle impact velocity, than the other models, especially for high speed impacts of over 30 km/h. These findings are similar to those from the analysis of thorax impact velocity (see Section 3.1). Generally, the predicted thoracic injury risks of the pedestrian in impacts with minivan model III were obviously higher than those with model I and model II, and the minivan model I (with extra bumper) produced slightly lower thoracic injury risks of the pedestrian than minivan model II for the higher speed cases. Specifically, all the minivan models resulted in quite low pedestrian thoracic injury risks (peak AIS3+ and AIS4+ injury risks of 22% and 5%, resp.) in impacts at a speed below 30 km/h, but the AIS3+ and AIS4+ injury risks substantially rose up to around 70% and 30%, respectively, for impacts at 50 km/h (see Figures 8 and 9).

## 4. Discussions

### 4.1. Pedestrian Kinematics and Chest Deformation

The effects of minivan front-end design and impact velocity on pedestrian kinematics were assessed by contact time of car to thorax region (CTCTR). As seen in Figure 5, for model III, compact engine compartment and high windshield slope produced a “flat” frontal surface, which meant that little time was needed by pedestrian thorax rotation before contacting with the vehicle. This was also a possible explanation on the prediction that the CTCTR of model III was the least sensitive to impact velocity. Furthermore, it could be imagined that for model III, the mild upper torso rotation and consequent early contact between the thorax and the vehicle would be helpful for spreading the impact loading to the whole body of the pedestrian and thus reducing the injury risks of the lower extremity. While for the other models, more time was needed to eliminate the relatively longer distance between pedestrian thorax and the vehicle by the rotation of pedestrian upper torso following a first contact between the lower extremity and the bumper, especially for the model II due to the extra bumper.

Figure 6 indicates that there was no big difference among the three minivan models when investigating how the vehicle velocity affected the thorax impact velocity, and that model I showed the lowest thorax impact velocities and model III shows the highest. However, for the chest deformation, it should be noted that the predicted chest deformation in impact with model III was slightly lower than that with model II (see Figure 7). Such finding was obviously not consistent with the predictions observed in Figure 6. This might imply that either thorax impact velocity or chest deformation is not a good predictor of human thoracic injury severity, although such investigation is not the purpose of our study.

### 4.2. Pedestrian Thoracic Injury Risk

As demonstrated in Figures 8 and 9, for all the vehicle impact velocity levels, the pedestrian sustained remarkably higher thoracic injury risks of both AIS3+ and AIS4+ in impacts with minivan model III than when struck by the remaining minivan models, especially at high impact velocities (40 and 50 km/h). This indicates that a flatter front minivan may increase the thoracic injury risk of the pedestrian. This finding is similar to those reported in the earlier studies [25, 27]. However, those earlier research work focused on comparing the minivan with other types of vehicles, while the current study particularly investigates how different minivan front shape in the market would affect the predicted pedestrian thoracic injury risk.

The pedestrian thoracic injury risk induced by model I (with extra bumper) was slightly lower than that by model II (without extra bumper), which implies that the application of the extra bumper would mitigate the pedestrian thoracic injury risks of both AIS3+ and AIS4+, but only resulting in minor difference, especially for impacts at a speed lower than 30 km/h. To the best of the authors' knowledge, such investigation has not been carried out before.

In the current study, TTI and chest deformation were used to represent the severity of thoracic injury; however, there are other injury parameters which were also used as thoracic injury predictors. It should be noted that all the injury parameters/criteria, employed in the current study or not, were proposed for the evaluation of vehicle crash safety regulations in terms of occupant protection rather than regarding the pedestrian injury assessment due to vehicle impact. Such particular injury parameter suitable for pedestrian thoracic injury evaluation is pended on further study.

### 4.3. Limitations of the Study

The limitations of this paper mainly rely on two aspects. The primary limitation is that the current study focuses on the evaluation of pedestrian thoracic injury risks, but in actual fact, pedestrian head and lower extremity pose more significant fatal and severe injuries, which has been identified in vast of related researches [3, 4, 15, 46, 47], and they were not considered in our study. Thus, from the perspective of pedestrian injury prevention involved in vehicle collisions, major research concerns need to be concentrated on the head and lower extremity injuries despite the importance/significance of thoracic injury inflicted when struck by minivan found both in the literature and in the current study.

Another limitation of this study is that the potential influence of the modifications of the minivan front-end shape, found to be conductive to reduce pedestrian thoracic injury risk in the current study, on the pedestrian lower extremity injury is not investigated. Since such influence has been analyzed in sedan-to-pedestrian impact accident scenarios [46, 48], further work may need to be conducted to identify it in the future research.

## 5. Conclusions

The current study reveals that the pedestrian may sustain a high thoracic injury risk when struck by a minivan. The results indicate that the analyzed parameters (including CTCTR, thorax impact velocity, and chest deformation) as well as the thoracic injury risks of the pedestrian are strongly affected by the variation of the minivan front-end design and the vehicle impact velocity. The main findings are as follows:

- CTCTR (the contact time of car to thorax region) of the pedestrian is more sensitive to the change of the minivan front-end design in low speed impacts than that in high speed impacts.
- The effects of vehicle impact velocity on thorax impact velocity and chest deformation are more appreciable than when changing minivan front-end design.
- The pedestrian thoracic injury risk increases with the increasing vehicle impact velocity, and the application of the extra front bumper is beneficial for reducing pedestrian thoracic injury risk.
- A relatively flatter minivan front-end, that is, the highest windshield slope in the current study (model III), may lead to a higher thoracic injury risk to pedestrian.
- The effects of minivan front-end design on pedestrian thoracic injury risks are particularly significant for high speed impacts (40 and 50 km/h), but such effects for low speed impacts (20 and 30 km/h) are much less important.

## Figures and Tables

**Table 1:** Calculated TTI values of the pedestrian when struck by the three minivan models at different impact velocities.

Impact velocity	20 km/h	30 km/h	40 km/h	50 km/h
Minivan model				
Model I	39.3	117.7	135.1	144.2
Model II	45.2	120.1	139.9	149.0
Model III	90.96	126.6	158.0	166.4

## Data Availability

The figures used to support the findings of this study are included within the article.

## References

[1] Forman J. L., Joodaki H., Forghani A. Whole-body response for pedestrian impact with a generic sedan buck.

[2] Zhao H., Yin Z., Chen R. (2010). Investigation of 184 passenger car–pedestrian accidents. *International Journal of Crashworthiness*.

[3] Wang B., Wang F., Otte D., Han Y., Peng Q. (2018). Effects of passenger car front profile and human factors on pedestrian lower extremity injury risk using German in-depth accident data. *International Journal of Crashworthiness*.

[4] Fahlstedt M., Halldin P., Kleiven S. (2016). Comparison of multibody and finite element human body models in pedestrian accidents with the focus on head kinematics. *Traffic Injury Prevention*.

[5] Naci H., Chisholm D., Baker T. D. (2009). Distribution of road traffic deaths by road user group: a global comparison. *Injury Prevention*.

[6] Li L., Yang J., Li W., Fang H., Zhao Z. (2005). A study on pedestrian injuries in traffic accidents. *Automotive Engineering*.

[7] Guo C., Chen K. (2006). Progress of epidemiological study on road traffic injuries in China. *Zhejiang Journal of Preventive Medicine*.

[8] Zhang Y., Lu H., Liu Q. (2006). The analysis of the road safety status and countermeasures in China. *Journal of Changsha Communications University*.

[9] Cai Z. (2009). *Finite Element Analysis of Bone Scathing of Lower Extremity in Car-Pedestrian Accidents*.

[10] Li K. (2015). *An In-Depth Investigation of Minibus Collisions and Study on Injury Mechanicsm*.

[11] Li K., Fan X., Yin Z. (2015). Pedestrian injury patterns and risk in minibus collisions in China. *Medical Science Monitor*.

[12] Isenberg R. A., Chidester A. B., Mavros S. Updated on the pedestrian crash data study.

[13] Maki T., Kajzer J., Mizuno K., Sekine Y. (2003). Comparative analysis of vehicle–bicyclist and vehicle–pedestrian accidents in Japan. *Accident Analysis & Prevention*.

[14] Mizuno Y. Editor summary of IHRA pedestrian safety working group activities-proposed test methods to evaluate pedestrian protection offered by passenger cars.

[15] Yang J., Otte D. (2007). *A comparison study on vehicle traffic accident and injuries of vulnerable road users in China and Germany*.

[16] Han Y., Yang J., Nishimoto K. (2012). Finite element analysis of kinematic behaviour and injuries to pedestrians in vehicle collisions. *International Journal of Crashworthiness*.

[17] Desapriya E., Subzwari S., Sasges D. (2010). Do light truck vehicles (LTV) impose greater risk of pedestrian injury than passenger cars? A meta-analysis and systematic review. *Traffic Injury Prevention*.

[18] Hu J., Klinich K. D. (2015). Toward designing pedestrian–friendly vehicles. *International Journal of Vehicle Safety*.

[19] Martin J.-L., Lardy A., Laumon B. Pedestrian injury patterns according to car and casualty characteristics in France.

[20] Fildes B., Gabler H. C., Otte D., Linder A., Sparke L. Pedestrian impact injuries using real-world crash data and harm.

[21] Chidester A. B., Isenberg R. A. Final report—the pedestrian crash data study.

[22] Li G., Nie J., Yang J. A study on injuries and kinematics in pedestrian accidents involved minivan and sedan.

[23] Liu W., Zhao H., Li K., Su S., Fan X., Yin Z. (2015). Study on pedestrian thorax injury in vehicle-to-pedestrian collisions using finite element analysis. *Chinese Journal of Traumatology*.

[24] Roudsari B. S., Mock C. N., Kaufman R. (2005). An evaluation of the association between vehicle type and the source and severity of pedestrian injuries. *Traffic Injury Prevention*.

[25] Tanno K., Kohno M., Ohashi N. (2000). Patterns and mechanisms of pedestrian injuries induced by vehicles with flat-front shape. *Legal Medicine*.

[26] Mizuno K., Kajzer J. Head injuries in vehicle-pedestrian impact.

[27] Han Y., Yang J., Mizuno K., Matsui Y. (2012). Effects of vehicle impact velocity, vehicle front-end shapes on pedestrian injury risk. *Traffic Injury Prevention*.

[28] LSTC (2007). *LS-DYNA Keyword User's Manual, Version 971*.

[29] Yao J., Yang J., Otte D. (2008). Investigation of head injuries by reconstructions of real-world vehicle-versus-adult-pedestrian accidents. *Safety Science*.

[30] Xu W., Yang J. (2008). Virtual test validation of human head model for injury assessment in traffic accidents. *Automotive Engineering*.

[31] Wang F., Xiao Z., Wan X., Yang J. (2010). FE modeling of the human neck responses in low-speed car collisions. *6th World Congress of Biomechanics (WCB 2010)*.

[32] Wang F., Yang J., Miller K. (2015). Numerical investigations of rib fracture failure models in different dynamic loading conditions. *Computer Methods in Biomechanics and Biomedical Engineering*.

[33] Wang F., Wang B., Han Y., Huang X., Yang J. (2017). A numerical study on correlation of rib fractures with thoracic injury criteria in oblique impact. *Journal of Mechanics in Medicine and Biology*.

[34] Han Y., Yang J., Mizuno K. (2011). Virtual reconstruction of long bone fracture in car-to-pedestrian collisions using multi-body system and finite element method. *Chinese Journal of Mechanical Engineering*.

[35] Yang J., Xu W., Wan X. (2005). Development and validation of a head-neck finite element model for the study of neck dynamic responses in car impacts. *Journal of Hunan University*.

[36] Li G., Yang J., Simms C. Predicting the effects of pedestrian gait on lower limb injuries.

[37] Han Y. (2011). *A Study on Dynamic Response and Injury Mechanism of Chest and Lower Extremities in Vehicle to Pedestrian Collisions*.

[38] Guo W. (2012). *A Study on Influences of Bull Bar Structures and Windshields on Pedestrian Injury Prevention*.

[39] Teresiński G., Mądro R. (2001). Knee joint injuries as a reconstructive factors in car-to-pedestrian accidents. *Forensic Science International*.

[40] Simms C. K., Wood D. P. (2006). Pedestrian risk from cars and sport utility vehicles-a comparative analytical study. *Proceedings of the Institution of Mechanical Engineers, Part D: Journal of Automobile Engineering*.

[41] Talantikite Y. B. Human thorax behaviour for side impact, influence of impact masses and velocities.

[42] Kuppa S., Eppinger R. H., McKoy F., Nguyen T., Pintar F. A., Yoganandan N. (2003). Development of side impact thoracic injury criteria and their application to the modified ES-2 dummy with rib extensions (ES-2re). *Stapp Car Crash Journal*.

[43] Tencer A. F., Kaufman R., Mack C., Mock C. (2005). Factors affecting pelvic and thoracic forces in near-side impact crashes: a study of US-NCAP, NASS, and CIREN data. *Accident Analysis & Prevention*.

[44] Eppinger R. H., Marcus J. H., Morgan R. M. Development of dummy and injury index for NHTSA's thoracic side impact protection research program.

[45] (2015). Regulation No 95 of the Economic Commission for Europe of the United Nations (UNECE): Uniform provisions concerning the approval of vehicles with regard to the protection of the occupants in the event of a lateral collision (2015/1093).

[46] Li G., Wang F., Otte D., Cai Z., Simms C. (2018). Have pedestrian subsystem tests improved passenger car front shape?. *Accident Analysis & Prevention*.

[47] Huang J., Long Y., Yan Y., Hu L. (2018). Development and validation of an age-specific lower extremity finite element model for simulating pedestrian accidents. *Applied Bionics and Biomechanics*.

[48] Li G., Lyons M., Wang B., Yang J., Otte D., Simms C. (2017). The influence of passenger car front shape on pedestrian injury risk observed from German in-depth accident data. *Accident Analysis & Prevention*.

